# The role of the gastrointestinal tract in phosphate homeostasis in health and chronic kidney disease

**DOI:** 10.1097/MNH.0b013e3283621310

**Published:** 2013-06-06

**Authors:** Joanne Marks, Edward S. Debnam, Robert J. Unwin

**Affiliations:** a London Epithelial Group, Department of Neuroscience, Physiology & Pharmacology; b UCL Centre for Nephrology, University College London Medical School, London, UK

**Keywords:** chronic kidney disease, intestine, NaPi-IIb, phosphate toxicity

## Abstract

**Purpose of review:**

For a number of years, there has been increasing interest in the concept of directly targeting intestinal phosphate transport to control hyperphosphatemia in chronic kidney disease. However, progress has been slow due to the paucity of information on the mechanisms involved in intestinal phosphate absorption. This editorial highlights the most recent developments in our understanding of this process and the role of the intestine in the maintenance of phosphate balance.

**Recent findings:**

Recent studies in *NaPi-IIb* knockout mice have confirmed that this transport protein plays a significant role in intestinal phosphate absorption and is critical in the proposed feed-forward mechanism between the small intestine and kidney, which helps to maintain normal phosphate balance and steady-state plasma phosphate concentrations. In addition, renal failure-induced hyperphosphatemia is attenuated in *NaPi-IIb* knockout mice, confirming that NaPi-IIb is a suitable target in the prevention and treatment of hyperphosphatemia.

**Summary:**

Recent findings suggest that consumption of processed foods containing phosphate preservatives may lead to excessive phosphate exposure (if not overload), toxicity, and cardiovascular disease in the general population, as well as in patients with declining renal function. Therefore, establishing more effective ways of targeting the intestine to limit dietary phosphate absorption could have wide-reaching health benefits.

## INTRODUCTION

There is mounting evidence that phosphate imbalance and altered homeostasis can result in calcium phosphate deposition in blood vessels, leading to stiffening of arteries and myocardial dysfunction, and an increased risk of cardiovascular disease. This form of ‘phosphate toxicity’ is widely recognized to occur in patients with chronic kidney disease (CKD), but it has also been hypothesized that consumption of food high in phosphate can cause similar cardiovascular changes in healthy individuals. Therefore, a better understanding of what controls phosphate balance has potentially wide-reaching benefits, particularly for patients with CKD.

Although there have been significant advances in our understanding of the hormonal changes that regulate phosphate balance and renal phosphate handling, our knowledge of the processes controlling intestinal phosphate absorption is still limited. This article reviews the recent literature on the role of the intestine in normal phosphate homeostasis and how this may be altered in CKD. It also highlights some new evidence for the concept of phosphate toxicity and the likely contribution from phosphate-containing additives used to flavor and preserve food.

## THE ROLE OF PHOSPHATE IN VASCULAR CALCIFICATION IN CHRONIC KIDNEY DISEASE

Hyperphosphatemia is a serious consequence of late stage CKD [1], leading to increased cardiovascular morbidity and mortality, particularly in patients on dialysis [2]. Normalization of serum phosphate levels has, therefore, been a clinical target in patients with CKD. However, as serum phosphate concentration represents a dynamic balance between intestinal phosphate absorption, renal phosphate excretion, and exchange between bone and extracellular storage pools, measurement of serum phosphate may not always be indicative of phosphate imbalance or total body phosphate load. Indeed, a recent short-term study of calcium and phosphate balance in patients with stage 3/4 CKD found no evidence for a significant net positive balance in phosphate (or calcium) [3]. That serum phosphate levels alone are not a good index of altered phosphate homeostasis is also highlighted by the finding that vascular calcification can occur in early CKD when phosphate levels are normal or near normal [4], and by the finding that fibroblast growth factor 23 (FGF-23) [5] and Klotho [6] may change even earlier in CKD, possibly independent of phosphate, and are potential predictors of vascular calcification and mortality in their own right. In addition, a recent study has demonstrated that even when serum phosphate levels are within the normal range, high serum FGF-23 concentrations combined with a low urinary fractional phosphate excretion are strongly associated with higher mortality and cardiovascular events, and that simultaneous measurement of these two parameters may also be predictors of patient outcome [7].

**Box 1:**
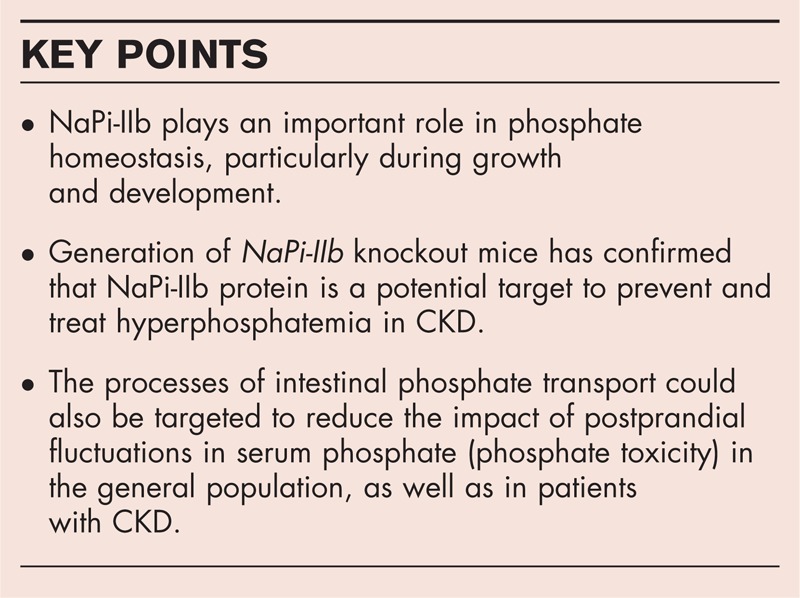
no caption available

Current strategies for the treatment of hyperphosphatemia in dialysis patients include dietary phosphate restriction and oral phosphate binders, although these treatments, if used aggressively, can lead to malnutrition, adverse gastrointestinal effects, and poor compliance with all medications, particularly in the elderly. Phosphate binders are effective at lowering serum phosphate levels, but they have recognized side-effects depending on their formulation, for example, aluminum toxicity and hypercalcemia. Efficacy and longer-term safety data on newer aluminum-free and calcium-free binders are becoming available and show that compounds such as sevelamer and lanthanum carbonate offer equivalent phosphate-lowering capacity and may reduce vascular calcification progression and improve patient outcomes (for a recent comprehensive review, see [8^▪^]).

Recent studies comparing different phosphate binders in patients with early and/or moderate CKD have reported lower mortality rates [9,10] and attenuated progression of secondary hyperparathyroidism, but increased progression of vascular calcification [11]. These findings highlight the need for more clinical trials before phosphate binders can be recommended for routine use in early or moderate CKD, but also demonstrate the need for alternative strategies for preventing phosphate imbalance. One such strategy under investigation is the targeted inhibition of the NaPi-IIb transporter; however, progress in this area has been hampered by the fact that our understanding of the control of intestinal phosphate transport *in vivo* is still quite limited.

## THE EMERGING CONCEPT OF DIET-INDUCED PHOSPHATE TOXICITY

There is now compelling evidence that phosphate is a risk factor for cardiovascular events in individuals with normal renal function [12,13] and that age-related cardiovascular changes may be a consequence of subtle changes in phosphate balance [14,15]. Indeed, studies have shown that healthy patients with serum phosphate more than 3.5 mg/dl (>1.13 mmol/l) have a 55% higher risk of developing cardiovascular disease [16].

Dietary phosphate consumption can vary significantly depending on food choices; ingestion of processed food containing high levels of phosphate preservatives may lead to supraphysiological postprandial spikes in blood phosphate levels and pose a long-term cardiovascular risk [17]. Consistent with this hypothesis is a recent study in healthy young women demonstrating that ingestion of two different phosphate salts commonly used as food additives resulted in significantly increased serum phosphate levels for up to 10 h, and that even after 20 h phosphate remained elevated [18^▪▪^]. These findings are particularly important for individuals on low incomes, which includes many patients with CKD, who are more than twice as likely to have hyperphosphatemia than those on higher incomes [19]. This difference is attributed to the high intake of cheaper processed food and is likely to pose a long-term cardiovascular risk in both healthy and CKD patients in this population.

## SOURCES OF DIETARY PHOSPHATE

Phosphate is present in high amounts in animal protein-based foods such as meat and fish, in dairy products, whole grains, and nuts. However, changes in the composition of our western diet have resulted in a dramatic, and almost hidden, increase in consumption of processed foods containing phosphate additives to enhance flavor, improve color, and to extend the shelf life of these products (see [20] for a comprehensive list of common phosphate additives used in food). A major concern is that the food industry is not currently required to provide information about naturally occurring or added phosphate levels in their food labeling; when this is given, the phosphate content is often underestimated or obscured by the complicated names of the different additives [21]. In fact, additives may increase the phosphate content of food by as much as 70% [22]. Another complicating factor is that inorganic phosphate from preservatives may have much higher bioavailability, resulting in more than 90% absorption, compared with only 40–60% for naturally occurring dietary phosphate [20].

## SODIUM-DEPENDENT VS. SODIUM-INDEPENDENT INTESTINAL PHOSPHATE ABSORPTION: INSIGHTS FROM *NaPi-IIb* KNOCKOUT MICE

Early studies showed that dietary phosphate absorption occurs in the small intestine [23,24] and that the underlying transport process could be resolved into sodium-dependent and sodium-independent components [25–27]. For a comprehensive overview of the older literature on phosphate transport and its regulation, see [28–30]. The realization that the gut is a potential target tissue for developing new therapeutic strategies to control hyperphosphatemia in CKD has led to more detailed investigation of the processes and regulation of intestinal phosphate transport. Targeted deletion of the *NaPi-IIb* gene has been shown to result in developmental arrest and fetal death [31,32], while conditional tamoxifen-inducible *NaPi-IIb*^−/−^ knockout mice [33] and heterozygote *NaPi-IIb*^+/−^ knockout mice [31] have been generated to investigate the role of this protein in intestinal phosphate transport and phosphate homeostasis postnatally. Table 1 summarizes the outcomes of studies using these mouse models.

Complete ablation of the *NaPi-IIb* gene has revealed that this protein accounts for approximately 90% of sodium-dependent phosphate transport across the mouse ileum [33]. However, this study also showed that even when this protein is maximally induced by feeding a low phosphate diet, the transporter accounted for only approximately 50% of total phosphate absorption, confirming early findings of a significant sodium-independent component of intestinal phosphate transport [26,27]. Adenine-induced CKD in *NaPi-IIb*^−/−^ knockout mice results in only partial prevention of hyperphosphatemia; the authors proposed that this might be a consequence of high passive transport rates of phosphate absorption caused by maintaining mice on a high phosphate diet [34^▪▪^]. In keeping with this explanation is the observation that treatment of CKD *NaPi-IIb*^−/−^ knockout mice with sevelamer normalized serum phosphate levels. Taken together, these findings clearly demonstrate that both sodium-dependent and sodium-independent phosphate transport occur in the small intestine and that both pathways can contribute to hyperphosphatemia in advanced CKD. Although our understanding of the role of NaPi-IIb in this process has increased through the generation of *NaPi-IIb*^−/−^ knockout mice, future studies are required to specifically investigate the mechanism(s) of sodium-independent phosphate transport, as this route is also a potential therapeutic target to limit hyperphosphatemia.

Reports to date suggest that the pathway for sodium-independent phosphate transport is unregulated [35–37]; however, it is unclear whether transport occurs via a transcellular or paracellular route. In this context, claudins and occludins are known to provide a paracellular route for passive ion flow, as well as providing cell adhesion between epithelial cells [38]. These tight junction proteins are regulated by signal transduction pathways and provide selectivity for paracellular ion transport. The finding that multiple isoforms of these proteins exist, with particular expression profiles along the gastrointestinal tract, suggests that they may have a specific role in controlling transport and barrier function in defined intestinal segments [38]. Whether tight junction proteins selectively influence paracellular phosphate transport has not been investigated. Alternatively, an unidentified transporter at the enterocyte brush border membrane (BBM) may be responsible for transcellular phosphate transport. In this regard, it is of interest to note the advancement in our understanding of the pathways involved in intestinal glucose absorption. Originally, the diffusive, sodium-independent component of intestinal glucose transport was attributed to paracellular transport [39]. However, it is now recognized that this pathway accounts for only 1–2% of glucose flux during a meal, and that the low affinity facilitative glucose transporter, GLUT2, provides the pathway for transcellular glucose absorption [40]. It is important to note that the facilitative pathway for intestinal glucose transport was overlooked in early in-vitro studies because of the rapid internalization of the GLUT2 away from the BBM during preparation of tissue for in-vitro uptake studies [41]. Therefore, using in-vivo techniques to examine the relevant contribution of these pathways to phosphate absorption may be more physiologically relevant.

## INTESTINAL TYPE III PHOSPHATE TRANSPORTERS

Studies have suggested that the type III sodium-dependent phosphate transporters, PiT1 and PiT2, are also involved in maintaining phosphate balance. In the rat, PiT1 is expressed at the enterocyte BBM and the regional expression mirrors that of NaPi-IIb [42]. In the mouse, PiT1 protein has been detected in both the proximal and distal small intestine [31], whereas so far PiT2 protein has been reported only in the ileum [43]. Studies have shown residual sodium-dependent phosphate transport in *NaPi-IIb*^−/−^ knockout mice accounting for approximately 10% of active transport; whether this represents PiT1-mediated or PiT2-mediated transport is unknown. Further studies will be necessary to determine whether these transporters make a significant contribution to intestinal phosphate absorption.

## PHOSPHATE HOMEOSTASIS: INSIGHTS FROM *NaPi-IIb* KNOCKOUT MICE

The heterozygote *NaPi-IIb*^+/−^ mouse has been used to highlight the function of this transporter in phosphate homeostasis during growth and development. At 4 weeks of age, *NaPi-IIb*^+/−^ mice show significant downregulation of NaPi-IIb mRNA and protein levels, with impaired intestinal phosphate transport and associated hypophosphatemia. Interestingly, the reduction in NaPi-IIb expression and function was also associated with decreased FGF-23 levels and activation of renal NaPi-IIa and NaPi-IIc transporter expression; however, renal adaptation was not sufficient to prevent hypophosphatemia [31]. In contrast, at 20 weeks, the reduced urinary phosphate excretion in *NaPi-IIb*^+/−^ mice was sufficient to maintain normal serum phosphate concentrations [31]. These findings support a critical role for NaPi-IIb in intestinal phosphate transport and phosphate homeostasis during ontogenesis, and confirm previous observations that sodium-dependent phosphate uptake and NaPi-IIb protein expression are highest during weaning, which reflects the higher phosphate requirement for normal skeletal growth [44,45].

Studies using *NaPi-IIb* knockout mice also highlight the possible role of NaPi-IIb in the proposed feed-forward mechanism linking the small intestine and kidney in maintaining phosphate balance. Altered urinary phosphate excretion and renal phosphate transporter expression are present in both *NaPi-IIb*^+/−^ and *NaPi-IIb*^−/−^ knockout mice, and are associated with decreased FGF-23 levels [31,34^▪▪^]. It is currently unclear whether these changes occur as a result of reduced intestinal phosphate transport or whether NaPi-IIb protein is part of the machinery responsible for triggering hormonal changes that influence phosphate balance. Of interest is the fact that the transporters responsible for the decrease in urinary phosphate excretion appear to differ depending on age and type of knockout mouse model used (see Table 1). The fact that NaPi-IIc is upregulated at 4 weeks in *NaPi-IIb*^+/−^ mice [31], but not at 10 weeks in *NaPi-IIb*^−/−^ mice [33] is perhaps not surprising, as NaPi-IIc expression and function in renal phosphate reabsorption has been reported to be age-dependent [46]. However, what is unexpected is that at 20 weeks, *NaPi-IIb*^+/−^ mice have persistent hypophosphaturia without changes in NaPi-IIa or NaPi-IIc expression, or FGF-23 levels [31]. The changes observed in renal phosphate transporter expression in mice with NaPi-IIb deletion might explain the finding that in human pulmonary alveolar microlithiasis, a disorder caused by inactivating mutations in NaPi-IIb, patients do not exhibit significant changes in serum phosphate concentrations [47], but do have decreased FGF-23 levels and urinary phosphate excretion [48].

## INTESTINAL PHOSPHATE TRANSPORT AND PHOSPHATE HOMEOSTASIS IN CHRONIC KIDNEY DISEASE

Our own research demonstrated that induction of CKD in rats, using the 5/6 nephrectomy model, did not alter intestinal phosphate transport, suggesting that the gut might be a suitable therapeutic target to prevent or reduce hyperphosphatemia [49]. A similar finding has been made in the Han:SPRD Cy/+ rat, a spontaneous model of renal cystic disease that develops CKD and mineral bone disorder. Contrary to the authors’ interpretation, the data show that jejunal phosphate flux in this model is unchanged when compared with normal rats on no binder treatment [50]. Moreover, studies in mice with adenine-induced renal failure also show BBM phosphate uptake is unchanged [31], and that there is either no change [31] or a nonsignificant trend for decreased NaPi-IIb protein levels following induction of CKD [34^▪▪^].

Recent mouse studies demonstrate for the first time that NaPi-IIb deletion attenuates hyperphosphatemia in models of CKD, supporting NaPi-IIb as a suitable treatment target for hyperphosphatemia in CKD. However, it is worth noting that induction of CKD in the two different mouse models of NaPi-IIb deletion had varying effects on serum phosphate levels. In the *NaPi-IIb*^+/−^ mouse, serum phosphate levels in adenine-induced CKD did not change significantly, whereas adenine-treated wild-type mice developed hyperphosphatemia [31]. In contrast, in the *NaPi-IIb*^−/−^ mouse there was only partial amelioration of hyperphosphatemia in adenine-induced CKD, and additional treatment with sevelamer was required to prevent this and reduce FGF-23 levels [34^▪▪^]. These inconsistencies have been attributed to differences in dietary phosphate bioavailability [34^▪▪^], but might also be due to inherent differences between the knockout mice *per se,* or in the mode and duration of adenine administration. Moreover, in *NaPi-IIb*^−/−^ mice with CKD induced surgically, rather than chemically, hyperphosphatemia is prevented [34^▪▪^]. Another interesting observation in adenine-treated *NaPi-IIb*^+/−^ mice was a decrease in serum creatinine levels, suggesting that reduced NaPi-IIb activity might also limit CKD progression; however, this apparent renoprotection was not seen in the *NaPi-IIb*^−/−^ mouse with adenine-induced or surgically induced renal failure, even when serum phosphate levels were normalized with binders [34^▪▪^]. Collectively, these findings suggest that development of inhibitors to directly target NaPi-IIb-mediated phosphate transport may prove therapeutically useful for at least some patients with CKD. However, it is envisaged that this treatment strategy will require the additional use of phosphate binders to effectively target both pathways of intestinal phosphate absorption, particularly in patients on dialysis. This combined approach might allow the use of lower doses of phosphate binders (which often cause significant gastrointestinal side-effects, leading to reduced patient acceptance and tolerance), as well reduce the risk of calcium overload when given as calcium carbonate [3], and it may enhance patient well being and lead to improved phosphate homeostasis.

## GUT–RENAL AXIS IN THE CONTROL OF PHOSPHATE HOMEOSTASIS

In 2007, a feed-forward mechanism between the small intestine and kidney was proposed in the regulation of phosphate balance. Instillation of phosphate into the duodenum released a putative ‘enteric phosphatonin’ that caused rapid renal excretion of phosphate. Intravenous infusion of a duodenal extract also evoked phosphaturia, demonstrating that the intestine was the probable source of this phosphatonin [51]. However, to date there has been no further published information on the mechanisms underlying this proposed entero-renal reflex or the identity of the putative phosphatonin, although it does not seem to be parathyroid hormone, FGF-23, or sFRP-4. However, as described earlier, there is considerable evidence from *NaPi-IIb* knockout mice supporting the concept that alterations in intestinal phosphate absorption induce rapid adaptive changes in renal phosphate handling to help maintain normal phosphate balance [31,33].

There is evidence that different regions of the small intestine may play distinct roles in phosphate homeostasis. In rat, the duodenum may be the locus of phosphatonin secretion [51], whereas the jejunum appears to be the major site of phosphate absorption and regulation [42,52,53]. Whether each intestinal region contains unique regulatory proteins specific to their roles in phosphate absorption has not been determined. It is, however, interesting to note that the phosphatonins FGF-23 (and its receptors, FGFR isoforms 1–4) [54] and matrix extracellular phosphoglycoprotein (MEPE) [29], and Klotho [55], are expressed in the small intestine, although their regional profile has not yet been examined in any detail. In addition, MEPE [53] and Klotho [55] are known to influence intestinal phosphate transport independent of FGF-23 or 1,25 dihydroxyvitamin D_3_, and may have local direct effects on phosphate absorption, as well as being candidates for the circulating enteric phosphatonin.

An interesting new concept is the potential role of the intestine in the regulation of circulating FGF-23 levels. The association between chronic hyperphosphatemia and FGF-23, and dietary phosphate intake and FGF-23, is well documented in both humans and animal models [56,57]. In contrast, acute modulation of serum phosphate levels, within the normal range, by nondietary intervention does not induce changes in FGF-23 [58,59]. Therefore, it appears that intestinal phosphate load and/or sensing may be the primary regulator of FGF-23 and that only supraphysiological changes in serum phosphate concentrations are associated with increased FGF-23 levels. However, how phosphate load is sensed by the intestine and what signal is responsible for stimulating FGF-23-secreting osteocytes is unknown and warrants further investigation.

## CONCLUSION

Tight control of phosphate balance and avoidance of phosphate overload are now recognized to have cardiovascular benefits for the general population, as well as patients with CKD. As increased dietary phosphate intake through consumption of processed foods poses a long-term risk to cardiovascular health, it is crucial to establish the contribution of different regions of the small intestine to dietary phosphate absorption, and to determine the mechanisms responsible for the interaction between gut and kidney in maintaining phosphate balance in health and disease. This information would enable us to develop more effective ways of manipulating intestinal phosphate transport to prevent wide fluctuations in serum phosphate levels, hyperphosphatemia, and phosphate overload in CKD.

## Acknowledgements

The authors gratefully acknowledge Kidney Research UK and the St Peter's Trust for Kidney, Bladder and Prostate Research for providing financial support for research described in this article.

### Conflicts of interest

J.M. has been an advisor to Ardelyx and AstraZeneca. R.J.U. is an external consultant to AstraZeneca, and has been an advisor to Amgen and Acologix. R.J.U. and E.S.D. have received research grants from Acologix. R.J.U. and J.M. have received a research grant from AstraZeneca.

## REFERENCES AND RECOMMENDED READING

Papers of particular interest, published within the annual period of review, have been highlighted as:

▪ of special interest
▪▪ of outstanding interest

Additional references related to this topic can also be found in the Current World Literature section in this issue (pp. 499–500).

## Figures and Tables

**Table 1:** Age and partial or complete ablation of the *NaPi-IIb* gene have different effects on parameters controlling phosphate balance

	4-week *NaPi-IIb*^+/−^	20-week *NaPi-IIb*^+/−^	10-week *NaPi-IIb*^−/−^
Intestinal sodium-dependent phosphate transport	↓ ∼50%	↓ ∼60%	↓ ∼90%
Urinary phosphate excretion	↓	↓	↓
NaPi-IIa protein expression	↑	↔	↑
NaPi-IIc protein expression	↑	↔	↔
Serum phosphate	↓	↔	↔
FGF-23	↓	↔	↓
1,25 dihydroxyvitamin D_3_	↑	↔	↑

FGF-23, fibroblast growth factor 23. Data from [29–32].

## References

[1] Indridason OS Quarles LD Hyperphosphatemia in end-stage renal disease. Adv Ren Replace Ther 2002; 9:184–192 12203200 10.1053/jarr.2002.34843

[2] Block GA Hulbert-Shearon TE Levin NW Port FK Association of serum phosphorus and calcium x phosphate product with mortality risk in chronic hemodialysis patients: a national study. Am J Kidney Dis 1998; 31:607–617 9531176 10.1053/ajkd.1998.v31.pm9531176

[3] Hill KM Martin BR Wastney ME Oral calcium carbonate affects calcium but not phosphorus balance in stage 3–4 chronic kidney disease. *Kidney Int* 2012 10.1038/ki.2012.403 [Epub ahead of print] PMC4292921 23254903

[4] Adeney KL Siscovick DS Ix JH Association of serum phosphate with vascular and valvular calcification in moderate CKD. J Am Soc Nephrol 2009; 20:381–387 19073826 10.1681/ASN.2008040349 PMC2637048

[5] Isakova T Xie H Yang W Fibroblast growth factor 23 and risks of mortality and end-stage renal disease in patients with chronic kidney disease. JAMA 2011; 305:2432–2439 21673295 10.1001/jama.2011.826 PMC3124770

[6] Lim K Lu TS Molostvov G Vascular Klotho deficiency potentiates the development of human artery calcification and mediates resistance to fibroblast growth factor 23. Circulation 2012; 125:2243–2255 22492635 10.1161/CIRCULATIONAHA.111.053405

[7] Dominguez JR Shlipak MG Whooley MA Ix JH Fractional excretion of phosphorus modifies the association between fibroblast growth factor-23 and outcomes. J Am Soc Nephrol 2013; 24:647–654 23520205 10.1681/ASN.2012090894 PMC3609138

[8▪] Frazao JM Adragao T Noncalcium-containing phosphate binders: comparing efficacy, safety, and other clinical effects. Nephron Clin Pract 2012; 120:c108–c119 22555359 10.1159/000337087

[9] Kovesdy CP Kuchmak O Lu JL Kalantar-Zadeh K Outcomes associated with phosphorus binders in men with nondialysis-dependent CKD. Am J Kidney Dis 2010; 56:842–851 20728255 10.1053/j.ajkd.2010.06.011 PMC2963702

[10] Di IB Bellasi A Russo D Mortality in kidney disease patients treated with phosphate binders: a randomized study. Clin J Am Soc Nephrol 2012; 7:487–493 22241819 10.2215/CJN.03820411

[11] Block GA Wheeler DC Persky MS Effects of phosphate binders in moderate CKD. J Am Soc Nephrol 2012; 23:1407–1415 22822075 10.1681/ASN.2012030223 PMC3402292

[12] Tonelli M Sacks F Pfeffer M Relation between serum phosphate level and cardiovascular event rate in people with coronary disease. Circulation 2005; 112:2627–2633 16246962 10.1161/CIRCULATIONAHA.105.553198

[13] Cancela AL Santos RD Titan SM Phosphorus is associated with coronary artery disease in patients with preserved renal function. PLoS One 2012; 7:e36883 22590632 10.1371/journal.pone.0036883 PMC3349637

[14] Razzaque MS Phosphate toxicity: new insights into an old problem. Clin Sci (Lond) 2011; 120:91–97 20958267 10.1042/CS20100377 PMC3120105

[15] Ohnishi M Razzaque MS Dietary and genetic evidence for phosphate toxicity accelerating mammalian aging. FASEB J 2010; 24:3562–3571 20418498 10.1096/fj.09-152488 PMC2923352

[16] Dhingra R Sullivan LM Fox CS Relations of serum phosphorus and calcium levels to the incidence of cardiovascular disease in the community. Arch Intern Med 2007; 167:879–885 17502528 10.1001/archinte.167.9.879

[17] Takeda E Yamamoto H Yamanaka-Okumura H Taketani Y Dietary phosphorus in bone health and quality of life. Nutr Rev 2012; 70:311–321 22646125 10.1111/j.1753-4887.2012.00473.x

[18▪▪] Karp HJ Kemi VE Lamberg-Allardt CJ Karkkainen MU Mono- and polyphosphates have similar effects on calcium and phosphorus metabolism in healthy young women. Eur J Nutr 2013; 52:991–996 22763799 10.1007/s00394-012-0406-5

[19] Gutierrez OM Anderson C Isakova T Low socioeconomic status associates with higher serum phosphate irrespective of race. J Am Soc Nephrol 2010; 21:1953–1960 20847142 10.1681/ASN.2010020221 PMC3014009

[20] Kalantar-Zadeh K Gutekunst L Mehrotra R Understanding sources of dietary phosphorus in the treatment of patients with chronic kidney disease. Clin J Am Soc Nephrol 2010; 5:519–530 20093346 10.2215/CJN.06080809

[21] Uribarri J Phosphorus additives in food and their effect in dialysis patients. Clin J Am Soc Nephrol 2009; 4:1290–1292 19608709 10.2215/CJN.03950609

[22] Benini O D’Alessandro C Gianfaldoni D Cupisti A Extra-phosphate load from food additives in commonly eaten foods: a real and insidious danger for renal patients. J Ren Nutr 2011; 21:303–308 21055967 10.1053/j.jrn.2010.06.021

[23] Walling MW Intestinal Ca and phosphate transport: differential responses to vitamin D3 metabolites. Am J Physiol 1977; 233:E488–E494 596443 10.1152/ajpendo.1977.233.6.E488

[24] Danisi G Murer H Inorganic phosphate absorption in small intestine. Handbook of physiology. The gastrointestinal system. Vol IV; 1991 323–336

[25] Borowitz SM Granrud GS Ontogeny of intestinal phosphate absorption in rabbits. Am J Physiol 1992; 262:G847–G853 1590395 10.1152/ajpgi.1992.262.5.G847

[26] Lee DB Walling MW Corry DB Phosphate transport across rat jejunum: influence of sodium, pH, and 1,25-dihydroxyvitamin D3. Am J Physiol 1986; 251:G90–G95 2425640 10.1152/ajpgi.1986.251.1.G90

[27] Borowitz SM Ghishan FK Phosphate transport in human jejunal brush-border membrane vesicles. Gastroenterology 1989; 96:4–10 2909436 10.1016/0016-5085(89)90757-9

[28] Murer H Forster I Biber J The sodium phosphate cotransporter family SLC34. Pflugers Arch 2004; 447:763–767 12750889 10.1007/s00424-003-1072-5

[29] Marks J Debnam ES Unwin RJ Phosphate homeostasis and the renal-gastrointestinal axis. Am J Physiol Renal Physiol 2010; 299:F285–F296 20534868 10.1152/ajprenal.00508.2009

[30] Sabbagh Y Giral H Caldas Y Intestinal phosphate transport. Adv Chronic Kidney Dis 2011; 18:85–90 21406292 10.1053/j.ackd.2010.11.004 PMC3071860

[31] Ohi A Hanabusa E Ueda O Inorganic phosphate homeostasis in sodium-dependent phosphate cotransporter Npt2b(+)/(−) mice. Am J Physiol Renal Physiol 2011; 301:F1105–F1113 21816756 10.1152/ajprenal.00663.2010

[32] Shibasaki Y Etoh N Hayasaka M Targeted deletion of the tybe IIb Na(+)-dependent Pi-co-transporter, NaPi-IIb, results in early embryonic lethality. Biochem Biophys Res Commun 2009; 381:482–486 19233126 10.1016/j.bbrc.2009.02.067

[33] Sabbagh Y O’Brien SP Song W Intestinal Npt2b plays a major role in phosphate absorption and homeostasis. J Am Soc Nephrol 2009; 20:2348–2358 19729436 10.1681/ASN.2009050559 PMC2799172

[34▪▪] Schiavi SC Tang W Bracken C Npt2b deletion attenuates hyperphosphatemia associated with CKD. J Am Soc Nephrol 2012; 23:1691–1700 22859851 10.1681/ASN.2011121213 PMC3458457

[35] Danisi G Bonjour JP Straub RW Regulation of Na-dependent phosphate influx across the mucosal border of duodenum by 1,25-dihydroxycholecalciferol. Pflugers Arch 1980; 388:227–232 6894191 10.1007/BF00658486

[36] Katai K Tanaka H Tatsumi S Nicotinamide inhibits sodium-dependent phosphate cotransport activity in rat small intestine. Nephrol Dial Transplant 1999; 14:1195–1201 10344361 10.1093/ndt/14.5.1195

[37] Katai K Miyamoto K Kishida S Regulation of intestinal Na+-dependent phosphate co-transporters by a low-phosphate diet and 1,25-dihydroxyvitamin D3. Biochem J 1999; 343:705–712 10527952 PMC1220605

[38] Amasheh S Fromm M Gunzel D Claudins of intestine and nephron: a correlation of molecular tight junction structure and barrier function. Acta Physiol (Oxf) 2011; 201:133–140 20518752 10.1111/j.1748-1716.2010.02148.x

[39] Madara JL Pappenheimer JR Structural basis for physiological regulation of paracellular pathways in intestinal epithelia. J Membr Biol 1987; 100:149–164 3430571 10.1007/BF02209147

[40] Kellett GL Brot-Laroche E Mace OJ Leturque A Sugar absorption in the intestine: the role of GLUT2. Annu Rev Nutr 2008; 28:35–54 18393659 10.1146/annurev.nutr.28.061807.155518

[41] Helliwell PA Richardson M Affleck J Kellett GL Stimulation of fructose transport across the intestinal brush-border membrane by PMA is mediated by GLUT2 and dynamically regulated by protein kinase C. Biochem J 2000; 350:149–154 10926838 PMC1221236

[42] Giral H Caldas Y Sutherland E Regulation of the rat intestinal Na-dependent phosphate transporters by dietary phosphate. Am J Physiol Renal Physiol 2009; 297:F1466–F1475 19675183 10.1152/ajprenal.00279.2009 PMC2781338

[43] Reining SC Liesegang A Betz H Expression of renal and intestinal Na/Pi cotransporters in the absence of GABARAP. Pflugers Arch 2010; 460:207–217 20354864 10.1007/s00424-010-0832-2

[44] Arima K Hines ER Kiela PR Glucocorticoid regulation and glycosylation of mouse intestinal type IIb Na-P(i) cotransporter during ontogeny. Am J Physiol Gastrointest Liver Physiol 2002; 283:G426–G434 12121891 10.1152/ajpgi.00319.2001

[45] Xu H Bai L Collins JF Ghishan FK Age-dependent regulation of rat intestinal type IIb sodium-phosphate cotransporter by 1,25-(OH)(2) vitamin D(3). Am J Physiol Cell Physiol 2002; 282:C487–C493 11832333 10.1152/ajpcell.00412.2001

[46] Segawa H Kaneko I Takahashi A Growth-related renal type II Na/Pi cotransporter. J Biol Chem 2002; 277:19665–19672 11880379 10.1074/jbc.M200943200

[47] Corut A Senyigit A Ugur SA Mutations in SLC34A2 cause pulmonary alveolar microlithiasis and are possibly associated with testicular microlithiasis. Am J Hum Genet 2006; 79:650–656 16960801 10.1086/508263 PMC1592565

[48] Olauson H Brandenburg V Larsson TE Mutation analysis and serum FGF23 level in a patient with pulmonary alveolar microlithiasis. Endocrine 2010; 37:244–248 20960258 10.1007/s12020-009-9299-3

[49] Marks J Churchill LJ Srai SK Intestinal phosphate absorption in a model of chronic renal failure. Kidney Int 2007; 72:166–173 17457376 10.1038/sj.ki.5002292

[50] Moe SM Radcliffe JS White KE The pathophysiology of early-stage chronic kidney disease-mineral bone disorder (CKD-MBD) and response to phosphate binders in the rat. J Bone Miner Res 2011; 26:2672–2681 21826734 10.1002/jbmr.485

[51] Berndt T Thomas LF Craig TA Evidence for a signaling axis by which intestinal phosphate rapidly modulates renal phosphate reabsorption. Proc Natl Acad Sci U S A 2007; 104:11085–11090 17566100 10.1073/pnas.0704446104 PMC1891094

[52] Marks J Srai SK Biber J Intestinal phosphate absorption and the effect of vitamin D: a comparison of rats with mice. Exp Physiol 2006; 91:531–537 16431934 10.1113/expphysiol.2005.032516

[53] Marks J Churchill LJ Debnam ES Unwin RJ Matrix extracellular phosphoglycoprotein inhibits phosphate transport. J Am Soc Nephrol 2008; 19:2313–2320 19005008 10.1681/ASN.2008030315 PMC2588094

[54] Khuituan P Teerapornpuntakit J Wongdee K Fibroblast growth factor-23 abolishes 1,25-dihydroxyvitamin D(3)-enhanced duodenal calcium transport in male mice. Am J Physiol Endocrinol Metab 2012; 302:E903–E913 22275752 10.1152/ajpendo.00620.2011

[55] Dermaku-Sopjani M Sopjani M Saxena A Downregulation of NaPi-IIa and NaPi-IIb Na-coupled phosphate transporters by coexpression of Klotho. Cell Physiol Biochem 2011; 28:251–258 21865732 10.1159/000331737

[56] Wolf M Update on fibroblast growth factor 23 in chronic kidney disease. Kidney Int 2012; 82:737–747 22622492 10.1038/ki.2012.176 PMC3434320

[57] Wolf M Forging forward with 10 burning questions on FGF23 in kidney disease. J Am Soc Nephrol 2010; 21:1427–1435 20507943 10.1681/ASN.2009121293

[58] Ito N Fukumoto S Takeuchi Y Effect of acute changes of serum phosphate on fibroblast growth factor (FGF)23 levels in humans. J Bone Miner Metab 2007; 25:419–422 17968495 10.1007/s00774-007-0779-3

[59] Burnett-Bowie SM Mendoza N Leder BZ Effects of gonadal steroid withdrawal on serum phosphate and FGF-23 levels in men. Bone 2007; 40:913–918 17157573 10.1016/j.bone.2006.10.016 PMC2083121

